# Efficacy and Safety of Daprodustat Vs rhEPO for Anemia in Patients With Chronic Kidney Disease: A Meta-Analysis and Trial Sequential Analysis

**DOI:** 10.3389/fphar.2022.746265

**Published:** 2022-03-10

**Authors:** Zhangning Fu, Xiaodong Geng, Kun Chi, Chengcheng Song, Di Wu, Chao Liu, Quan Hong

**Affiliations:** ^1^ Medical School of Chinese PLA, Beijing, China; ^2^ Department of Nephrology, State Key Laboratory of Kidney Diseases, Beijing Key Laboratory of Kidney Diseases, Chinese PLA General Hospital, Chinese PLA Institute of Nephrology, National Clinical Research Center for Kidney Diseases, Beijing, China

**Keywords:** daprodustat, anemia, chronic kidney disease, meta-analysis, trial sequential analysis

## Abstract

**Introduction:** Daprodustat, a novel hypoxia-inducible factor prolyl-hydroxylase inhibitor (HIF-PHI), its efficacy and safety remain unclear. Thus, we conducted this meta-analysis aiming at investigating its efficacy and safety on the treatment of patients with chronic kidney disease (CKD)-related anemia.

**Methods:** We systematically searched for relevant studies in PubMed, Embase, Cochrane Library and Clinical Trial Registries databases from inception until December 2021. We selected randomized controlled trials comparing daprodustat with recombinant human erythropoietin (rhEPO) in anemia patients with CKD with or without dialysis.

**Results:** Seven studies including 7933 patients met the inclusion criteria. For both nondialysis-dependent (NDD-) CKD and dialysis-dependent (DD-) CKD patients, the pooled results showed that there was no significant difference in the changes in hemoglobin levels between the daprodustat and rhEPO groups (mean difference (MD) = −0.01, 95% confidence interval (CI) = −0.38, 0.35, *p* = 0.95; MD = 0.15, 95% CI = −0.29, 0.60, *p* = 0.50; respectively). In addition, a significant increase in transferrin saturation (TSAT), total iron binding capacity (TIBC) and total iron was observed in daprodustat groups compared with rhEPO groups in DD-CKD patients (*p* < 0.05). As for safety, the overall frequency of adverse events was similar between the daprodustat and rhEPO groups in DD-CKD patients (relative risk (RR) = 0.99, 95%CI = 0.92, 1.06, *p* = 0.76), and the trial sequential analysis (TSA) confirmed this result. But for NDD-CKD patients, the incidence of adverse events in the daprodustat groups was significantly higher than that of rhEPO groups (RR = 1.04, 95%CI = 1.01,1.07, *p* = 0.02), while the TSA corrected this result. No trend of increasing incidence of serious adverse events was found in all daprodustat treated patients, but the TSA could not confirm this result.

**Conclusion:** Although daprodustat was noninferior to rhEPO in correcting anemia in both NDD-CKD and DD-CKD patients, it seemed to have a better effect on optimizing iron metabolism in DD-CKD patients. Daprodustat may be a promising alternative for the treatment of anemia in patients with CKD. However, due to the lack of included studies, future researches are needed to further evaluate the therapeutic effect of daprodustat.

**Systematic Review Registration:** https://www.crd.york.ac.uk/prospero/, identifier CRD42021229636.

## Introduction

Anemia is a common complication in millions of patients with progressive chronic kidney disease (CKD) and is associated with poor clinical outcome in dialysis-dependent (DD-) and nondialysis-dependent (NDD-) CKD patients (Thomas et al., 2008). Anemia of CKD is caused by multiple factors and is mainly the result of the impaired kidney being unable to adequately respond to hypoxia and/or anaemia by inducing erythropoietin (EPO) production (Babitt and Lin, 2012). Other factors include reduced iron availability, infection and inflammation (Bonomini et al., 2016). Recombinant human erythropoietin (rhEPO) or its analogs (erythropoiesis-stimulating agents [ESAs]) and iron supplementation (intravenous and/or oral) represent the current standard of treatments for CKD patients with anemia (Drüeke and Parfrey, 2012). However, their use in correcting anemia also has some limitations due to its inconvenience of injection and some existing safety concerns (Bonomini et al., 2016). There is evidence that application of high ESA dose is associated with increased risk of stroke, hypertension, cardiovascular events and all-cause mortality (Koulouridis et al., 2013, Locatelli et al., 2013; Bonomini et al., 2016). Iron supplementation also has some significant drawbacks. Intravenous (IV) iron has potential adverse impacts on stimulating bacterial growth, increasing the risk of infection, and direct cellular toxicity (Fishbane et al., 2004; Kalantar-Zadeh et al., 2009). Excessive iron use also does harm to the human body (Hung and Tarng, 2014).

Daprodustat (GSK1278863), a novel small-molecule hypoxia-inducible factor (HIF) prolyl hydroxylase inhibitor (PHI) which belongs to an emerging new therapeutic class of agents is currently being developed by GlaxoSmithKline (GSK) for the treatment of anaemia in patients with CKD (Dhillon, 2020). Daprodustat can inhibit HIF-prolyl hydroxylase domain enzymes (PHD1, PHD2, and PHD3), which leads to the accumulation of HIF-α transcription factor and altered expression of HIF-responsive genes (Gupta and Wish, 2017). And as a result, it stimulates erythropoiesis and improves the iron metabolism (Haase, 2013). Researchers have investigated the efficacy and safety of daprodustat in recent years. Although pervious meta-analyses (Xie et al., 2018; Zheng et al., 2020a) have shown that daprodustat could improve hemoglobin and is well tolerated in CKD patients, the evidence for its efficacy and safety in NDD patients and DD patients is still lacking. Therefore, we performed this meta-analysis and trial sequential analysis (TSA) to further evaluate the efficacy and safety of daprodustat for the treatment of CKD-associated anemia in both NDD and DD patients.

## Methods

The Preferred Reporting Items for Systematic Reviews and Meta-Analyses (PRISMA statement) guidelines were applied to perform this meta-analysis (Moher et al., 2009). This meta-analysis was previously registered on PROSPERO database (Registration number: CRD42021229636).

### Data Sources and Study Selection

Electronic databases including PubMed, Embase, the Cochrane Library and Clinical Trial Registries databases were searched from inception to December 2021, using items related to “daprodustat,” and “GSK1278863”. The search was limited to studies involving human subjects, and no language restrictions were applied. The citations of the included studies were scanned to identify additional relevant studies if necessary.

### Inclusion and Exclusion Criteria

The inclusion criteria were as follows: (Thomas et al., 2008) study design: randomized controlled trials (RCTs); (Babitt and Lin, 2012) population: anemia patients with CKD (>18 years old) with or without dialysis; (Bonomini et al., 2016) intervention: daprodustat compared with rhEPO (epoetins or their biosimilars or darbepoetin); and (Drüeke and Parfrey, 2012) outcome: assessed at least one of the following outcomes: the change in hemoglobin, hepcidin, transferrin saturation (TSAT), ferritin, total iron-binding capacity (TIBC) and total iron, IV iron therapy rate, adverse events (nonfatal events such as gastrointestinal discomfort, hypertension and hypotension) and serious adverse events (fatal events such as cardiac failure, acute renal failure and sepsis) during the treatment. The exclusion criteria were as follows: (Thomas et al., 2008) studies that involved healthy individuals; (Babitt and Lin, 2012) studies that included inappropriate comparisons or did not include a reference group; (Bonomini et al., 2016) studies with research data that could not be extracted and analyzed.

### Data Extraction and Quality Assessment

Two reviewers (ZF and CL) independently extracted the data using a standardized form. Each trial was assessed using the Cochrane risk of bias tool. The standard criteria included the following domains: random sequence generation, allocation concealment, blinding of participants and personnel, blinding of outcome assessment, incomplete outcome data, and selective reporting and other bias. Any disagreements were solved by a third reviewer. The extracted data included first author, year of publication, patient characteristics, sample size, doses of treatment, control, the outcomes of adverse events, serious adverse events, change in hemoglobin level, hepcidin, transferrin saturation (TSAT), ferritin, total iron-binding capacity (TIBC), total iron and IV iron therapy rate.

### Data Synthesis and Statistical Analysis

All data were analyzed using Review Manager (version 5.3, The Cochrane Collaboration, Oxford, United Kingdom). The effect size was assessed by relative risks (RRs) with 95% confidence intervals (CIs) for dichotomous outcomes and mean differences (MDs) with 95% CIs for continuous outcomes. Heterogeneity across the trials was assessed using the I^2^ statistic, and I^2^ > 50% indicated significant heterogeneity (Higgins et al., 2003). Subgroup analysis was conducted to investigate between-study heterogeneity. All P-values were two-sided, and a P-value less than 0.05 was considered to indicate a statistically significant difference. If the mean or standard deviation of the outcomes could not be directly extracted from the studies, we estimated them from the sample size, median, range and/or interquartile range (Hozo et al., 2005; Wan et al., 2014).

### Trial Sequential Analysis

Trial sequential analysis (TSA) was used in the meta-analyses to lower the risk of obtaining a false-positive or false-negative conclusion (Brok et al., 2008) and was performed by using TSA Version 0.9.5.10 Beta (www.ctu.dk/tsa). A sufficient level of evidence for the anticipated intervention effect was reached and no further trials were needed if the cumulative Z-curve crossed the trial sequential monitoring boundary or entered the futility area, whereas if the Z-curve did not cross any of the boundaries or the required information size (RIS) has not been reached, the evidence of the conclusion was considered to be insufficient and more trials were needed to confirm the results (Liu et al., 2016). For this TSA, we estimated the RIS based on a RR reduction of 15% with a power (1-β) of 0.85 for adverse events and a RR reduction of 20% with a power (1-β) of 0.80 for serious adverse events. The type I error (*α*) = 0.05 (two-sided). The control event proportion was calculated from the comparator group (Liu et al., 2019).

## Results

### Study Enrollment and Study Characteristics

**FIGURE 1:**
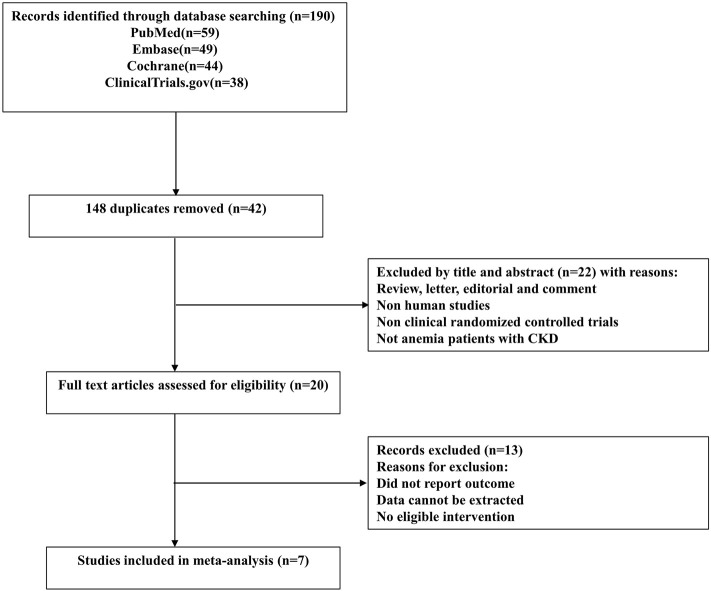
Flow diagram of the study selection process for the meta-analysis.

The study selection process is depicted as a flow diagram in Figure 1. After the removal of duplicates and studies that failed to meet the inclusion criteria, 20citations were retrieved for detailed assessment. 13 articles were excluded because they did not include a reference group or the comparisons were inappropriate. In total, 7 RCTs (Holdstock et al., 2016; Holdstock et al., 2019; Meadowcroft et al., 2019; Akizawa et al., 2020; Singh et al., 2021a; Singh et al., 2021b; Nangaku et al., 2021) involving 7933 participants were included in this meta-analysis. Four studies (Akizawa et al., 2020; Singh et al., 2021a; Singh et al., 2021b; Nangaku et al., 2021) were phase 3 clinical trials, and the other three (Holdstock et al., 2016; Holdstock et al., 2019; Meadowcroft et al., 2019) were phase 2 clinical trials. The characteristics of component trials and patient demographic data are summarized in Table 1. These trials were reported between 2016 and 2021. The follow-up for patients ranged from 4 to 52 weeks.

**TABLE 1:** Characteristics of included studies.

Source	Country	NCT number	Setting	Intervention	Type of patients	Duration	Iron therapy	No. of patients (M/F)	Mean age, years
Akizawa T et al. (2020)	Japan	02969655	Phase 3, randomized, double-blind, active-controlled, parallel-group	T: Daprodustat (started with 4 mg and adjusted every 4 weeks within the range of 1–24 mg) once daily for 52 weeks	DD-CKD	52 weeks	Oral or IV	T: 136 (91/45)	T: 64 ± 10a
				C: rhEPO (Darbepoetin alfa: started at a corresponding dose to the prior rhEPO and then adjusted every 2 weeks within the range of 10–60 μg) IV once weekly				C: 135 (89/46)	C: 64 ± 11a
Holdstock L et al. (2016)	6 countries (United States, Canada	01587924	Phase 2, randomized, triple-blind, active-controlled, parallel-group, multicenter	T: Daprodustat (0.5, 2 or 5 mg) once daily for 4 weeks	DD-CKD	4 weeks	Oral	T: 62 (43/19)	T: 55.7 ± 17.4a
	Germany, Denmark, Norway, Sweden)			C: rhEPO (dose: NA)				C: 20 (16/4)	C: 64.2 ± 12.8a
Holdstock L et al. (2019)	15 countries	01977573	Phase 2, randomized, open-label, active-controlled, parallel-group, multicenter	T: Daprodustat (1, 2 or 4 mg) once daily for 24 weeks	NDD-CKD	24 weeks	Oral	T: 156 (64/92)	T: 66.5 ± 12.78a
				C: rhEPO (dose for every participant was determined by the investigator)				C: 79 (33/46)	C: 65.4 ± 13.6a
Meadowcroft A et al. (2019)	16 countries	01977482	Phase 2, randomized, triple-blind (open-label rhEPO), dose-ranging, active-controlled, parallel-group, multicenter	T: Daprodustat (4, 6, 8, 10 or 12 mg) once daily for 24 weeks	DD-CKD	24 weeks	Oral or IV	T: 171 (108/63)	T: 59.6 ± 13.3a
				C: Placebo for 4 weeks and then open-label rhEPO (dose for every participant was determined by the investigator to achieve hemoglobin within the target range (10–11.5 g/dl) as required)				C: 39 (26/13)	C: 59.7 ± 18.7a
Nangaku M et al. (2021)	Japan	02791763	Phase 3, randomized, open-label, active-controlled, parallel-group, multicenter	T: Daprodustat (1, 2, 4, 6, 8, 12, 18 or 24 mg) once daily for 52 weeks, C: rhEPO (Epoetin beta pegol: started at the dose of 25 μg every 2 weeks for rhEPO-naïve patients and 25–250 μg every 4 weeks for rhEPO users and then adjusted every 4 weeks)	NDD-CKD	52 weeks	Oral or IV	T: 149 (96/53)	T: 68 ± 12a
								C: 150 (92/58)	C: 70 ± 9a
Singh A et al. (2021)	38 countries	02876835	Phase 3, randomized, open-label, active-controlled, parallel-group, multicenter	T: Daprodustat (started between 1 and 4 mg and then adjusted within the range of 1–24 mg) once daily for 52 weeks	NDD-CKD	52 weeks	Oral or IV	T: 1937 (835/1102)	T: 67 (57–75)b
				C: rhEPO (Darbepoetin alfa: started based on the patient’s weight and hemoglobin level at the time of randomization for rhEPO-naïve patients or on the previous dose for rhEPO users. Dose stepped changes were predefined, and most steps increased the dose by 25–33%)				C: 1935 (864/1071)	C: 67 (57–74)b
Singh A et al. (2021)	35 countries	02879305	Phase 3, randomized, open-label, active-controlled, parallel-group, multicenter	T: Daprodustat (started between 4 and 12 mg and then adjusted within the range of 1–24 mg) once daily for 52 weeks	DD-CKD	52 weeks	Oral or IV	T: 1487 (851/636)	T: 58 (48–67)b
				C: rhEPO (started based on the previous dose and hemoglobin level at the time of randomization. Dose stepped changes were predefined, and most steps increased the dose by 25–33%.)				C: 1477 (847/630)	C: 59 (47–68)b

Abbreviations: C, control group; CKD, chronic kidney disease; DD, dialysis-dependent; IV, intravenous; M/F, male/female; NCT, national clinical trial; NDD, non-dialysis-dependent; rhEPO, recombinant human erythropoietin; T, treatment group; TIW, three times weekly; aMean±standard deviation.

### Quality Assessment of the Included Studies

**FIGURE 2:**
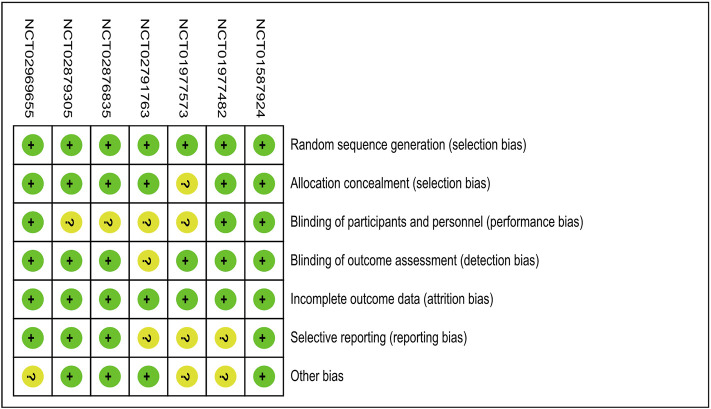
Assessment of risk of bias. Abbreviation: NCT, National Clinical Trial.

The details for the risk of bias tool are shown in Figure 2. Review Manager 5.3 was used to evaluate the overall quality of the articles. All studies have a low risk of selection bias.

### Effects on Hemoglobin Level

**FIGURE 3:**
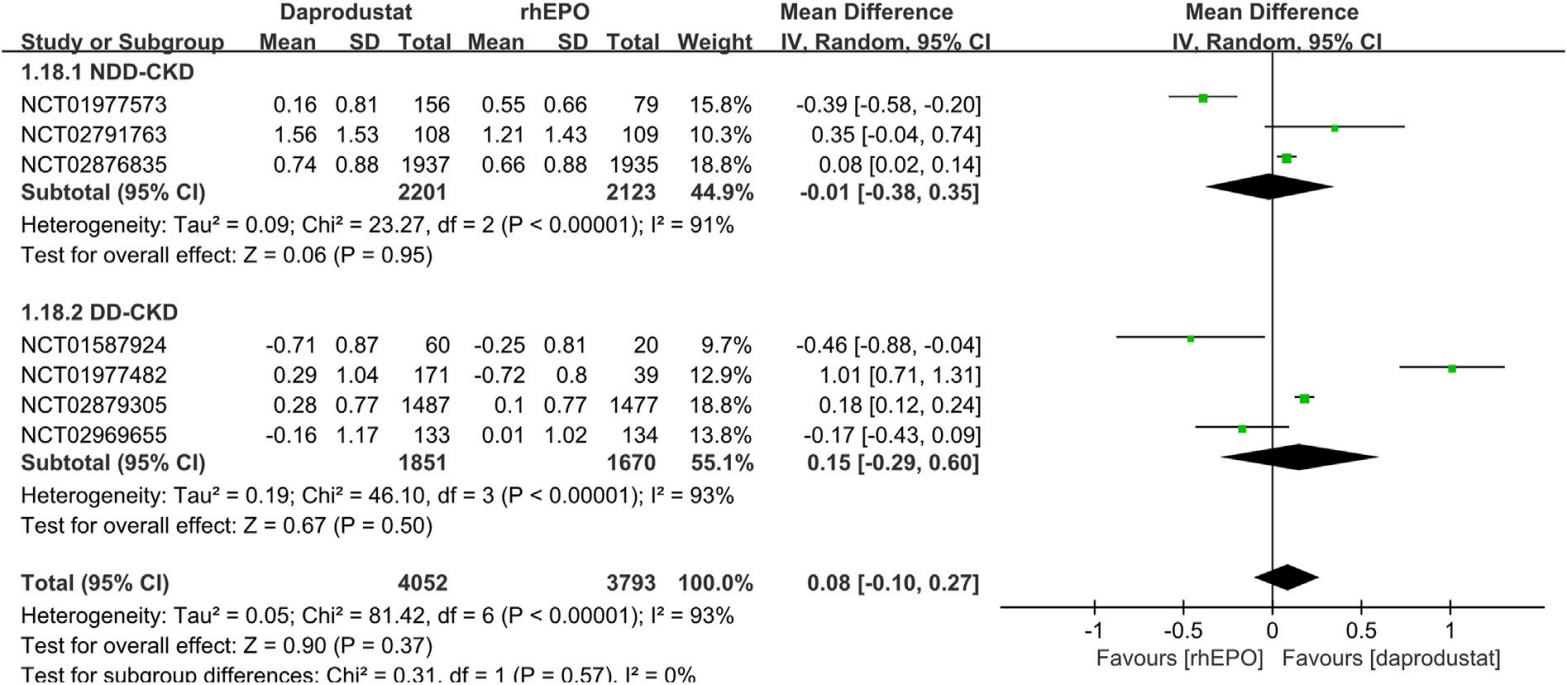
Comparison of the change in hemoglobin level between daprodustat and rhEPO. Notes: NDD-CKD: MD = −0.01, 95% CI = −0.38, 0.35, *p* = 0.95, I^2^ = 91%; DD-CKD: MD = 0.15, 95% CI = −0.29, 0.60, *p* = 0.50, I^2^ = 93%.

Seven studies (Holdstock et al., 2016; Holdstock et al., 2019; Meadowcroft et al., 2019; Akizawa et al., 2020; Singh et al., 2021a; Singh et al., 2021b; Nangaku et al., 2021) made comparisons of changes in hemoglobin level and six studies (Holdstock et al., 2016; Holdstock et al., 2019; Meadowcroft et al., 2019; Akizawa et al., 2020; Singh et al., 2021a; Nangaku et al., 2021) reported the number of patients who met the protocol-defined hemoglobin stopping criteria. In both NDD-CKD and DD-CKD patients, daprodustat showed an effect similar to that of rhEPO on increasing the hemoglobin level (Figure 3). A larger number of patients were found to discontinue the therapy in daprodustat group for reaching the predefined lower hemoglobin threshold, but the difference was not significant (Table 2).

**TABLE 2:** Other outcomes.

Outcomes	Subgroup	Number of studies	Mean difference or risk ration (95%CI)	Test for effect (*p* value)	Heterogeneity
					I^2^ (*p* value)
Discontinuation rate	NDD-CKD	3 [24,26,27]	1.53 [0.27, 8.86]	0.63	52% (0.12)
	DD-CKD	3 [22,23,25]	2.93 [0.53, 16.06]	0.22	0% (0.60)
△Hepcidin (ng/ml)	NDD-CKD	3 [24,26,27]	−32.62 [−54.75, −10.49]	<0.01	90% (< 0.01)
	DD-CKD	4 [22,23,25,28]	−9.26 [−33.69, 15.16]	0.46	87% (< 0.01)
△Ferritin (ng/ml)	NDD-CKD	3 [24,26,27]	−20.18 [−31.73, −8.64]	<0.01	0% (0.76)
	DD-CKD	4 [22,23,25,28]	−22.80 [−61.94, 16.34]	0.25	76% (< 0.01)
△Total iron-binding capacity (ug/dL)	NDD-CKD	3 [24,26,27]	29.00 [19.65, 38.35]	<0.01	70% (0.04)
	DD-CKD	4 [22,23,25,28]	33.85 [25.06, 42.64]	<0.01	76% (< 0.01)
△Transferrin saturation (%)	NDD-CKD	3 [24,26,27]	-9.71 [−19.94, 0.52]	0.06	48% (0.14)
	DD-CKD	4 [22,23,25,28]	2.64 [0.31, 4.97]	0.03	0% (0.92)
△Total iron (ug/dL)	NDD-CKD	3 [24,26,27]	−2.10 [−3.71, −0.49]	0.01	0% (0.59)
	DD-CKD	4 [22,23,25,28]	11.46 [9.74, 13.19]	<0.01	0% (0.48)
IV iron therapy rate	NDD-CKD	2 [24,26]	1.72 [0.79, 3.75]	0.17	0% (0.72)
	DD-CKD	2 [22,25]	0.88 [0.65, 1.20]	0.42	59% (0.12)
Outcomes	Subgroup	Number of studies	Value	Study	Maximum observed VEGF^b^	Test for effect (*p* value)
VEGF levels (ng/L)	NDD-CKD	1 [24]	T: 65.8 (17.9, 467.9)^a^	[24]	T:105.8 (33.1,1205.3)	>0.05
			C: 68.7 (22.9, 1118.5)^a^		C:94.5 (25.5, 472.7)	
	DD-CKD	2 [23,25]	T: 2.03 ± 65.63^b^	[23]	NA	>0.05
			C: 1.2 ± 40.3^b^	[25]	T: 270.0 (81.8, 808.3)	>0.05
			T: 186.9 (64.3, 1142.6)^a^		C: 269.4 (98.7, 924.0)	
			C: 220.0 (78.2, 595.7)^a^			

Abbreviations: C, control group; CI, confidence interval; CKD, chronic kidney disease; DD, dialysis-dependent; IV, intravenous; NA, not available; NDD, non-dialysis-dependent; rhEPO, recombinant human erythropoietin; T, treatment group; VEGF, vascular endothelial growth factor; a baseline, median (minimum, maximum); b change from baseline (mean ± SD).

### Effects on Iron Metabolism Parameters

The outcomes of iron metabolism, including changes in hepcidin, TSAT, ferritin, TIBC and total iron are investigated in all the studies. In NDD-CKD patients (Holdstock et al., 2019; Nangaku et al., 2021; Singh et al., 2021a), daprodustat significantly decreased the level of hepcidin, ferritin, total iron and increased the level of TIBC compared with rhEPO, while no significant difference of TSAT was observed between the two groups. In DD-CKD patients (Holdstock et al., 2016; Meadowcroft et al., 2019; Akizawa et al., 2020; Singh et al., 2021b), daprodustat significantly increased TSAT, TIBC and the total iron compared with rhEPO, but hepcidin and ferritin levels were not obviously changed. In addition, four studies (Holdstock et al., 2019; Meadowcroft et al., 2019; Akizawa et al., 2020; Nangaku et al., 2021) reported the results of iron administration modes and dosages used in rhEPO versus daprodustat treated patients during the treatment. The pooled results showed that no significance was found in IV therapy rate between the daprodustat and rhEPO groups in both NDD and DD patients (Table 2). But for iron dosages, the specific data could not be extracted and pooled for analysis.

### Safety of Daprodustat

**FIGURE 4:**
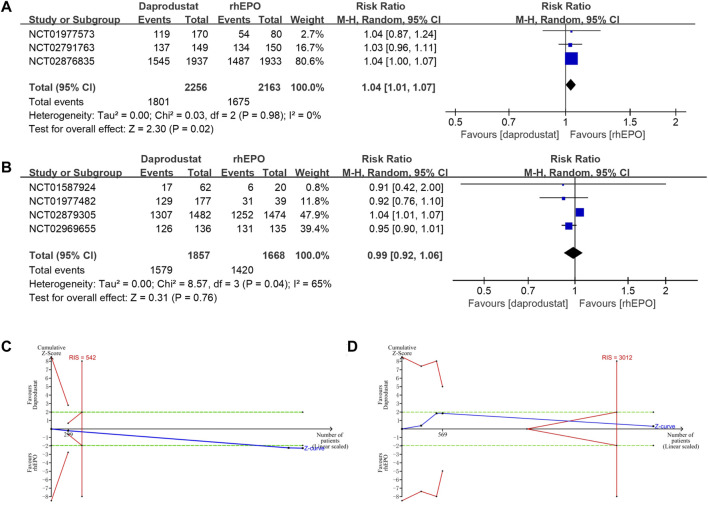
The adverse events of daprodustat. **(A)** Forest plot of the adverse events of daprodustat and rhEPO in NDD-CKD patients. Notes: RR = 1.04, 95% CI = 1.01, 1.07, *p* = 0.02, I^2^ = 0%. **(B)** Forest plot of the adverse events of daprodustat and rhEPO in DD-CKD patients. Notes: RR = 0.99, 95% CI = 0.92, 1.06, *p* = 0.76, I^2^ = 65%. **(C)** Random effects model of the TSA of adverse events of daprodustat and rhEPO in NDD-CKD patients. A diversity-adjusted information size of 542 participants was calculated based on an adverse event rate of 77.4% in the rhEPO group and a RR reduction of 15%, with α = 5% (two-sided), β = 15%, and I^2^ = 0%. The solid blue line represents the cumulative Z-curve, which crossed the futility boundary (solid red line). **(D)** Random effects model of the TSA of adverse events of daprodustat and rhEPO in DD-CKD patients. A diversity-adjusted information size of 3012 participants was calculated based on an adverse event rate of 85.1% in the rhEPO group and a RR reduction of 15%, with α = 5% (two-sided), β = 15%, and I^2^ = 65%. The solid blue line represents the cumulative Z-curve, which crossed the futility boundary (solid red line).

**FIGURE 5:**
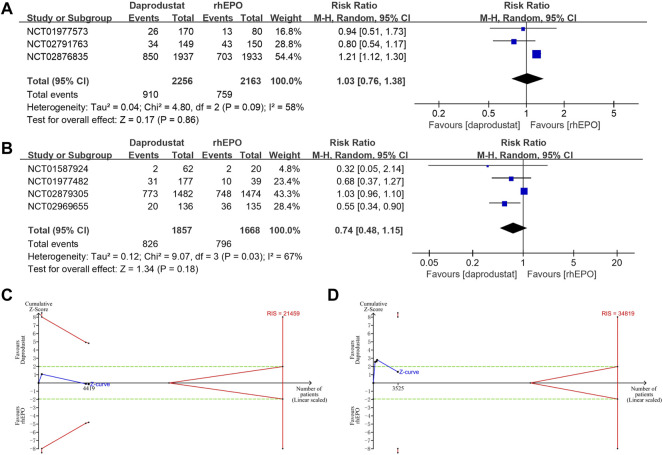
The serious adverse events of daprodustat. **(A)** Forest plot of the serious adverse events of daprodustat and rhEPO in NDD-CKD patients. Notes: RR = 1.03, 95% CI = 0.76, 1.38, *p* = 0.86, I^2^ = 58%. **(B)** Forest plot of the serious adverse events of daprodustat and rhEPO in DD-CKD patients. Notes: RR = 0.74, 95% CI = 0.48, 1.15, *p* = 0.18, I^2^ = 67%. **(C)** Random effects model of the TSA of serious adverse events of daprodustat and rhEPO in NDD-CKD patients. A diversity-adjusted information size of 21459 participants was calculated based on an adverse event rate of 35.1% in the rhEPO group and a RR reduction of 20%, with α = 5% (two-sided), β = 20%, and I^2^ = 58%. The solid blue line represents the cumulative Z-curve, which did not cross the conventional boundary (dashed green line) and the trial sequential monitoring boundary (solid red line). **(D)** Random effects model of the TSA of serious adverse events of daprodustat and rhEPO in DD-CKD patients. A diversity-adjusted information size of 34819 participants was calculated based on an adverse event rate of 47.7% in the rhEPO group and a RR reduction of 20%, with α = 5% (two-sided), β = 20%, and I^2^ = 67%. The solid blue line represents the cumulative Z-curve, which did not cross the futility boundary (solid red line).

All studies reported adverse events and serious adverse events of the treatment during follow-up (22–28). The use of daprodustat was associated with a higher risk of adverse events than rhEPO in patients with NDD-CKD (Figure 4A), but this result was rectified by the TSA, as the cumulative Z curve crossed the futility boundary and reached the required information size (Figure 4C). No significant difference was noted between daprodustat and rhEPO in terms of adverse events in patients with DD-CKD (Figure 4B), and the TSA confirmed this result, for the cumulative Z-curve crossed the futility boundary and entered the futility area (Figure 4D). The overall frequency of serious adverse events was similar between the daprodustat and rhEPO groups in both NDD-CKD and DD-CKD patients (Figures 5A,B), but the TSA suggested that the evidence to reach this conclusion was insufficient, because the cumulative Z-curve did not cross the futility boundary and enter the futility area (Figures 5C,D).

## Discussion

We performed this meta-analysis to evaluate the efficacy and safety of daprodustat versus rhEPO in anemic patients with NDD-CKD or DD-CKD. The results indicated that daprodustat is well tolerated in CKD patients and has an effect similar to that of rhEPO on increasing hemoglobin. Furthermore, the changes in the iron metabolism parameters suggested that daprodustat has the ability to improve iron utilization in DD-CKD patients, however, in NDD-CKD patients, this result cannot be concluded. Therefore, additional clinical trials are desired to find out whether daprodustat can optimize iron metabolism in NDD-CKD patients.

Previous meta-analyses have shown that daprodustat could improve hemoglobin level without increasing the incidence of adverse events and serious adverse events in the short term compared with rhEPO. However, the evidence for its safety is still inadequate. In contrast with those meta-analyses, we further used a TSA to provide a more sufficient and conclusive evidence for adverse events in daprodustat and compared the data of VEGF in daprodustat with rhEPO.

Daprodustat (GSK1278863), a once-daily oral novel small-molecule HIF-PHIs, is a new class of agents being developed by GlaxoSmithKline (GSK) for anemic patients with CKD (10). It has the ability to inhibit HIF-prolyl hydroxylase domain enzymes (PHD1, PHD2, and PHD3), and ultimately resulting in stimulating the expression of erythropoietin genes, up-regulating endogenous erythropoietin (EPO) levels and optimizing iron bioavailability (Beuck et al., 2012; Haase, 2013; Gupta and Wish, 2017; Kaplan et al., 2018). In this process, HIF2α plays a key role in mediating the cellular response to hypoxia and in turn regulates the intestinal iron uptake, iron transport and the use of iron *via* hepcidin-dependent and hepcidin-independent mechanisms (Koury and Haase, 2015; van Swelm et al., 2019). Hepcidin here is a cationic antimicrobial peptide (CAPs) that plays a key role in the regulation of iron metabolism and is a mediator of anemia of inflammation (Ganz, 2003). It not only impairs the iron absorption from the duodenal enterocytes but also the iron release from macrophages, where most iron is stored (Provenzano et al., 2016). Hepcidin has a major role in the anemia of CKD and elevated hepcidin level is likely to contribute to the incidence and severity of anemia, thus agents that have an effect of lowering hepcidin or inhibiting its actions may be effective on correcting anemia of CKD (Coyne, 2011). Similar to HIF-PHIs, rhEPO also has a positive influence on iron metabolism. It can affect iron homeostasis directly by increasing the transferrin receptor expression and iron uptake into erythroid cells (Busfield et al., 1997; Weiss et al., 1997). So, it is of great importance to make a comparison on the effect of iron metabolism between daprodustat and rhEPO.

According to the evidence from this meta-analysis, daprodustat may indeed have an effect on regulating iron metabolism in DD-CKD patients (Holdstock et al., 2016; Meadowcroft et al., 2019; Akizawa et al., 2020). Daprodustat significantly increased the level of TSAT, TIBC and serum iron compared with rhEPO. TSAT and TIBC are both efficacious biomarkers for the diagnose of iron deficiency anemia. TIBC has acted as a surrogate nutritional marker in multiple studies (Kalantar-Zadeh et al., 1998; Bross et al., 2009), and TIBC levels may be low in multifactorial anemias or anemias of chronic inflammation (Faruqi and Mukkamalla, 2021). In addition, lower TIBC also predicted poorer prognosis in both postoperative and hemodialysis patients (Bross et al., 2009; Sawayama et al., 2018). TSAT was also proposed as an alternative or complementary diagnostic test for iron deficiency. TSAT reflects iron availability for erythropoiesis. And the decrease of TSAT is one of the earliest biomarkers of iron deficiency, whether absolute or functional (Peyrin-Biroulet et al., 2015). Among CKD patients, TSAT ≤20% could be found in patients with absolute iron deficiency or functional iron deficiency (also known as iron-restricted erythropoiesis) (Gafter-Gvili et al., 2019). Also, studies have shown that low TSAT levels are related to an increased risk of cerebrovascular and cardiovascular disease (CCVD) and death compared to patients with normal or higher TSAT levels (Kuragano et al., 2020). Therefore, the increasing level of TSAT, TIBC and total iron suggested that daprodustat may be superior to rhEPO in optimizing iron metabolism for the treatment of anemia in DD-CKD patients. As for NDD-CKD patients, the use of daprodustat significantly reduced the levels of hepcidin and ferritin as well as serum total iron, suggesting that the body’s iron storage and available iron were decreased after the treatment. Besides, no tendency of decreased IV therapy rate was detected in both NDD and DD patients. Due to the lack of iron application standard in daprodustat clinical studies, it is difficult to evaluate the truly effect of daprodustat on iron metabolism. Therefore, further investigations concerning the effects of daprodustat on iron mobilization with larger-sample sizes, more standardized criteria and longer-duration time are required.

In view of the results of C-reactive protein levels in previous clinical trials, it was found that there was a difference in inflammation level between NDD and DD patients and DD patients were more likely to be associated with a higher level of inflammation which had a non-ignorable effect on the management of CKD-related anemia (Eustace et al., 2004; Costa et al., 2008; Holdstock et al., 2016; Watanabe et al., 2016; Singh et al., 2021a; Singh et al., 2021b). Thus, the efficacy of daprodustat in patients with inflammation status is worth to be examined. A study showed that patients with rhEPO hyporesponsiveness who were treated with daprodustat received less IV iron than those who were treated with rhEPO (Singh et al., 2021b). This result suggests that daprodustat may exert a better effect on iron availability for erythropoiesis when used in inflamed patients compared with rhEPO. This finding may be of great significance since rhEPO resistance is strongly correlated with inflammation and in this situation the effect of rhEPO will be decreased (Perunicic-Pekovic et al., 2008). Moreover, as shown in the results presented above, iron parameters were different in NDD and DD patients after the treatment of daprodustat or rhEPO, indicating that the effect of daprodustat or rhEPO may be different between NDD and DD patients. In light of the different degrees of inflammatory states in NDD and DD patients, the different levels of these iron parameters may be attributed to the different levels of inflammation. Thus, inflammation might be responsible for these contrasting differences between the two regimens in NDD-CKD and DD-CKD patients. However, owing to the fact that the relevant data were not given in our included studies or could not be extracted, we were not able to conduct an analysis to compare whether there was indeed a difference between daprodustat and rhEPO treatments in iron availability for erythropoiesis and in iron homeostasis in patients with or without inflammation. It should also be noticed that both daprodustat and rhEPO have the ability to up-regulate plasma EPO which has an anti-inflammatory effect leading to the differences in circulating iron parameters (Nairz et al., 2011). Hence, additional trials are needed to further compare the effect of daprodustat and rhEPO on patients under the condition of inflammation and determine which approach possess a better anti-inflammatory ability.

In this meta-analysis, we also used a TSA to provide more conservative estimates and to establish sufficient and conclusive evidence of adverse events. Although an increased risk of adverse events was found in daprodustat groups versus the control groups in NDD-CKD patients, TSA corrected this result, indicating that within the set assumptions for confidence and effect size, daprodustat intervention is not related with higher relative risk of harm in comparison with rhEPO. For DD-CKD patients, we found sufficient evidence that there was no significant difference in the adverse events associated with daprodustat compared with rhEPO, and the TSA confirmed this result. No trend of increasing incidence of serious adverse events was observed in all daprodustat treated subjects. However, the TSA could not prove this result. Since the HIF-PHI pathway is involved in multiple biological processes, such as the upregulation of the erythropoietin gene, promotion of tumor metastasis by stimulating epithelial-to-mesenchymal transition (Yang et al., 2008) and induction of tumor cell invasion (Meijer et al., 2012), safety concerns for HIF stabilizers including the risk of the development or progression of malignancy, diabetic retinopathy, heart failure, pulmonary hypertension, autoimmune disease, kidney fibrosis and polycystic kidney disease should be carefully concerned (Koury and Haase, 2015). Considering that patients with CKD-associated anemia are all chronic patients that need long-term medication and HIF-PHIs is a kind of agents that is highly likely to exhibit off-target activity in injured kidneys (Hasegawa et al., 2018), additional trials with long observation periods focused on the safety of daprodustat are urgently needed.

In addition, three studies (Holdstock et al., 2016; Holdstock et al., 2019; Meadowcroft et al., 2019) evaluated the vascular endothelial growth factor (VEGF) expression of the patients during the treatment process (Table 2). However, the data extracted from the studies cannot be merged to analyze for their different forms of expression. The effect of daprodustat on plasma VEGF concentration was determined in these studies because of the HIF-prolyl hydroxylase inhibitors have a potential ability to increase VEGF levels through induction of the VEGF gene (Asikainen et al., 2005). Furthermore, HIF–prolyl hydroxylases may indirectly regulate VEGF through their effects on EPO, which in turn stimulates the production of VEGF (Nitta et al., 1999). VEGF is a homodemeric disulfide bound glycoprotein that promotes endothelial growth, accompanied by higher vascular permeability (Theis and Theiss, 2018). And it is now well-known that VEGF is essential for physiologic vascular homeostasis in various cells and tissues, and has been demonstrated to be critical in the molecular pathogenesis of tumor growth and metastasis and in retinopathy associated with several blinding eye diseases, including age-related macular degeneration (AMD) and diabetic and hypertensive retinopathy (Adamis and Shima, 2005; Ferrara, 2016; Melincovici et al., 2018). VEGF mediates these progresses mainly via its effects on vascular permeability and neoangiogenesis (neovascularization). Thus, the observation of the changes in plasma VEGF concentrations during the treatment period is important. In the three studies (Holdstock et al., 2016; Holdstock et al., 2019; Meadowcroft et al., 2019) that have reported the comparisons of plasma VEGF concentrations in daprodustat versus rhEPO in both NDD-CKD and DD-CKD patients, there was no clinically significant elevations in plasma VEGF concentrations (Table 2).

The efficacy and safety of other HIF-PHIs, such as roxadustat (FG-4592), molidustat (BAY85-3934), vadadustat (AKB-6548), enarodustat (JTZ-951) and desidustat in the treatment of CKD-related anemia has also been investigated by several clinical trials. According to recent meta-analyses (Wang et al., 2020; Zheng et al., 2020b), desidustat seems to have the highest potential in elevating hemoglobin level and daprodustat ranked fifth among the six included HIF-PHIs. Vadadustat is the least potent HIF-PHIs. And for iron homeostasis, daprodustat still does not seem to have the best ability in down regulating the hepcidin level. But the conclusion is certainly not sufficient since no study has directly compared the efficacy and safety among these drugs so far. Therefore, additional clinical and preclinical studies are needed to determine which agent has the highest efficacy and lowest toxicity.

### Limitations

There are several limitations in our meta-analysis. Firstly, the number of studies included in this analysis is small and the data for some of the results are scarce, therefore, we could only provide preliminary results on the safety and efficacy of daprodustat. Secondly, three phase 2 trials are included in this meta-analysis, which decrease the level of evidence. Thirdly, the dosage of daprodustat is varied in the included RCTs, and several phase-II studies have demonstrated that daprodustat exerts a dose-dependent effect on hemoglobin, thus we were not able to evaluate its most efficacious and safest dose in both NDD and DD patients. Fourthly, long-term follow-up results are not yet available, so the long-term effect of daprodustat cannot be investigated. Fifthly, iron supplementation is not consistent among the included studies which may influence the results of the comparisons between daprodustat and rhEPO.

## Conclusion

In conclusion, our meta-analysis showed that daprodustat was noninferior to rhEPO in rectifying anemia in both NDD-CKD and DD-CKD patients. However, daprodustat may better optimize iron metabolism in dialysis patients with renal anemia. Daprodustat may be a promising alternative for the management of anemia in patients with CKD. Additional clinical trials are still needed to further validate the value of daprodustat.

## Data Availability

The original contributions presented in the study are included in the article/Supplementary Material, further inquiries can be directed to the corresponding authors.
